# Prize Essays for 1853

**Published:** 1852-12

**Authors:** 


PRIZE ESSAYS FOR 1853.

It will be seen from the following circular that two prizes of the value of $100 each are to be awarded by the American Medical Association at its next annual meeting, to the two best essays that may be offered.

*American Medical Association.*—At the meeting of the Association held at Richmond, Va., May 1852, the undersigned were appointed a committee to receive voluntary communications on medical subjects, and to award two prizes of $100 each to the authors of the best two essays.

Each communication must be accompanied by a sealed packet, containing the name of the author, which will be opened only in the case of the successful competitors. Unsuccessful communications will be returned on application after June 1st, 1853.

Communications must be addressed post-paid, to the chairman of the committee, Dr. Joseph M. Smith, 56 Bleecker street, New York, on or before the 20th of March, 1853.

